# Engineering terpene synthases and their substrates for the biocatalytic production of terpene natural products and analogues

**DOI:** 10.1039/d4cc05785f

**Published:** 2024-12-28

**Authors:** Luke Alan Johnson, Rudolf Konrad Allemann

**Affiliations:** a School of Chemistry, Cardiff University, Main Building, Park Place CF10 3AT Cardiff UK AllemannRK@cardiff.ac.uk

## Abstract

Terpene synthases produce a wide number of hydrocarbon skeletons by controlling intramolecular rearrangements of allylic pyrophosphate subtrates *via* reactive carbocation intermediates. Here we review recent research focused on engineering terpene synthases and modifying their substrates to rationally manipulate terpene catalyisis. Molecular dynamic simulations and solid state X-ray crystallography are powerful techniques to identify substrate binding modes, key active site residues for substrate folding, and the location of active site water. Variants in specific ‘hotspots’ of terpene synthases including the G1/2, K/H and Hα-1 helices have been targeted to modify active site water management and yield new products. We discuss the potential of exploiting substrate analogues to synthesise novel compounds and briefly outline biphasic flow systems for biocatalysis of terpenes. We forsee greater applications for terpenes as the field converges on effective methods for engineering of terpene synthases by new computational and high throughput experimental methods and for high-yield production. It is crucial when engineering terpene synthases that both product distribution and enzyme activity are simultaneously optimised.

## Abstract 2

Summarises recent contributions to rationally engineer terpene synthase catalysis to control water capture and utilise substrate analogues to produce terpene natural products and their analogues.

## Graphical abstract

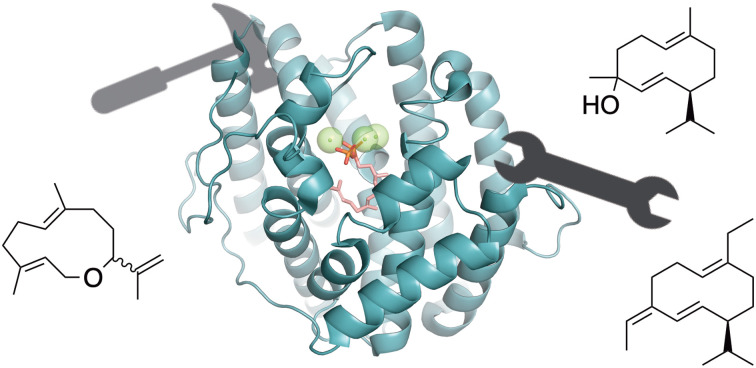

## 1. Introduction

Nature has established remarkable chemical diversity of terpenoid natural products by varying enzyme activity and combining enzyme functions. More than 80 000 natural terpenoids have been characterised;^1^ many have applications as pharmaceuticals, agrochemicals, fragrances, cosmetics, flavourings, or biofuels. For instance, paclitaxel is a key anticancer chemotherapy,^2^ artemisinin is a first-line antimalarial treatment,^3^ and squalene is used as an emollient in cosmetics and an adjuvant in vaccines.^4^ Although terpene natural products are frequently valuable, they are often produced by natural sources in low quantities and are mostly a challenge to synthesise in the laboratory by conventional synthetic chemistry methods due to their chemical and stereochemical complexity; often they have limited stability to temperature, light, oxygen or acidic conditions.^5^ For paclitaxel, artemisinin and squalene the natural quantities originally limited applications, and alternative sources or increases in production were needed.^6–9^ Bioproduction by either heterologous expression in alternative host organisms or by *in vitro* biocatalysis offer attractive routes to produce terpenoids if the enzymes governing the biosynthesis and the chemistry are available. By harnessing non-natural pathways, substrates and enzymes, and by manipulating enzyme activities, there is potential to expand the natural scope of terpene chemistry and drive the production of novel terpenoids with new activities. Alongside others our recent research has focused on establishing predictive methods for modifying terpene synthase catalysis and applying it to the production of both natural and non-natural terpenes.

### 1.1. Biosynthesis of terpenoids

**Fig. 1:**
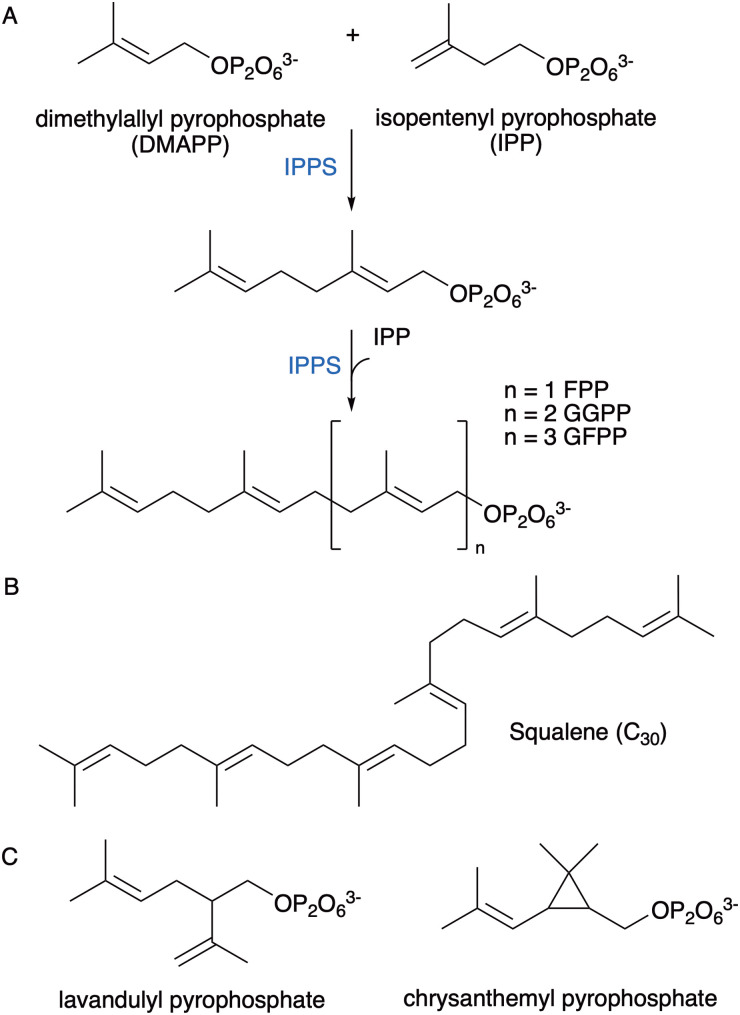
Biosynthesis of terpene precursors from DMAPP and IPP. (A) Regular ‘head-to-tail’ chain extension to C10 GPP and higher order prenyl pyrophosphates. (B) Structure of squalene formed from presqualene diphosphate (PSDP), the ‘head-to-head’ condensation of two FPP molecules, and the subsequent NADPH-dependent reductive rearrangement of PSDP. (C) Example of irregular ‘head-to-middle’ coupling products derived from two DMAPP molecules.

All terpenoids are derived from dimethylallyl pyrophosphate (DMAPP) and isopentenyl pyrophosphate (IPP). ‘Head-to-tail’ condensation of DMAPP and IPP by prenyl transferases (PT) catalyse the formation of C10 geranyl pyrophosphate (GPP; monoterpenes) and with further sequential additions of IPP, C15 farnesyl pyrophosphate (FPP; sesquiterpenes) and C20 geranylgeranyl pyrophosphate (GGPP; diterpenes) (Fig. 1A).^10^ Extra IPP additions produce further higher order terpenoids, while ‘head-to-head’ condensations of FPP or GGPP coupled to a reductive step produce squalene and phytoene, the precursors to tri- and tetraterpenoids (Fig. 1B).^11–13^ Other, irregular terpenoids can be synthesised from two DMAPP molecules by ‘head-to-middle’ condensations (Fig. 1C).^14^ Non-canonical terpenes have atypical number of carbon atoms (*e.g.* C11, C16, C17), and can be synthesised through methyltransferase-catalysed methylation of prenyl pyrophosphates.

Terpene synthases catalyse the often complex reaction cascades of linear prenyl pyrophosphates substrates to acyclic or cyclic terpene products.^1,15^ Catalysis involves initial carbocation formation by either loss of pyrophosphate or protonation. After ionisation, a series of controlled intramolecular rearrangements occur including ring closures, hydride and proton shifts, and methyl migrations. Finally, deprotonation or quenching of the carbocation with water in the terpene synthase active site leads to neutral terpene hydrocarbons or oxygenated products. Occasionally, the neutral product can be protonated to form a further carbocation from which additional cyclisation and rearrangement reactions progress.^16,17^ Terpene products can be functionalised to terpenoids through a wide variety of modifying enzymes including cytochrome P450s,^18,19^ epoxidases,^20^ acyltransferases,^21–23^ methyltransferases^24,25^ and glycosyltransferases.^26^

Although terpenoids are present in all forms of life, the diversity of natural products is not uniformly distributed. Plants produce a wide array of volatile mono- and sesqui-terpenes for defence and as semiochemicals.^27^ The tomato (*Solanum lycopersicum* L.) has more than 30 terpene synthases in its genome.^28,29^ Genome sequencing has provided a wealth of terpene synthase sequences from a wide assortment of organisms and led to a growing number of established activities.^30–34^ Unfortunately, mining genomes for novel activities suffers from challenges with predicting terpene products based on primary sequence alone. Low sequence homology across terpene synthases from different organisms limits the potential to extrapolate activities and although some degree of prediction is possible including using the active site volume and N-terminal targeting sequences to forecast substrate preference, product prediction usually requires experimental testing.^33,35–37^ If the chemistry of terpene synthase catalysis can be better understood for predictive synthesis, then ‘designer’ terpene synthases may be possible which manipulate the reaction coordinate to produce novel terpenes as well as natural terpenes for which the terpene synthase has not been identified or the natural enzymes are not suitable for production.

## 2. Engineering terpene synthase catalysis

### 2.1. Terpene synthases

There are two classes of terpene synthases, class I and II. For class I enzymes two conserved metal binding motifs (the aspartate-rich DDXX(X)D motif and the DTE/NSE triad motif ND(L,I,V)XSXXXE) bind three magnesium ions, leading to loss of pyrophosphate and generation of the initial carbocation, while class II terpene synthases catalyse the protonation of the substrate through a general acid.^15,38,39^ In both classes, the active site geometry drives the cyclisation cascade towards products through steric arrangement of the substrate and electrostatic control of the carbocation intermediates.^40,41^ The active site surrounding the substrate hydrocarbon chain is chiefly arranged with nonpolar aliphatic and aromatic residues with low reactivity towards carbocation intermediates creating a largely inert active site. Aromatic residues stabilise carbocations through π-carbocation interactions and steer the formation of specific carbocations intermediates, including examples where the carbocation positive charge preferentially forms on less substituted carbons of the substrate (anti-Markovnikov addition).^42,43^

The structure of class I and II terpene synthases are distinct.^1,15^ For class I enzymes, the active site is located within the centre of an α helical bundle, defined as the α domain. For class II the active site is within a cleft formed from two alpha helical β and γ domains, likely derived from gene duplication.^44^ Domain arrangements can vary between enzymes (αβγ, α, αα, αβ, βγ) and can include either class I, class II or both activities.^1^

### 2.2. Terpene synthase cyclisation

Terpene synthases can have high fidelity and produce a single major product with only minor side products or produce a variety of similarly populated products from different reaction pathways and carbocation intermediates. For instance, bornyl pyrophosphate synthase produces α-pinene, camphene, limonene, and terpinolene as side products to bornyl pyrophosphate.^45^ Terpene synthases can even have different selectivity for substrate chain lengths and stereochemistry.^46–50^ For low fidelity enzymes the steric control of the substrate provided by the active site contour is presumably less clearly defined and multiple binding geometries accommodated. Predicting substrate and intermediate binding modes is demanding as the hydrocarbon substrate chain lacks clearly defined interactions such as hydrogen bonds and instead bind to the terpene synthase active site pocket through hydrophobic interactions, van der Waals contacts and weak electrostatics.^51^ Computational methods that use consensus docking methods have been developed to help predict substrate, intermediate and transition state binding modes.^37^

**Fig. 2:**
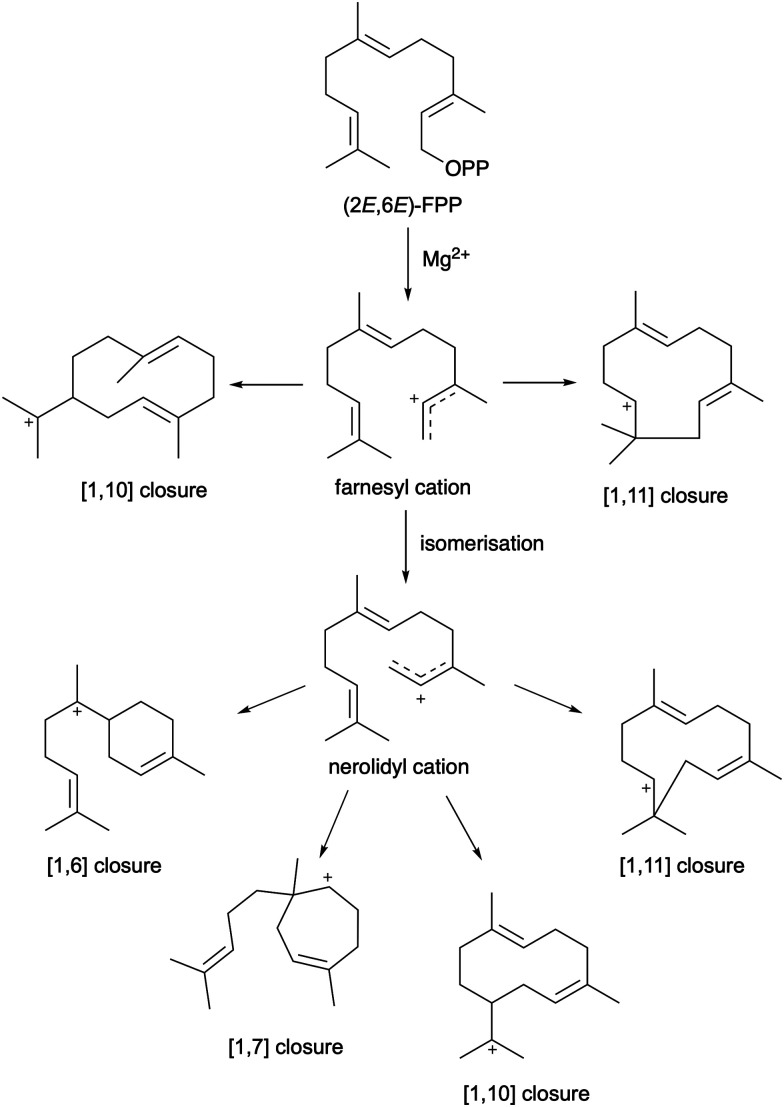
Examples of sesquiterpene cyclisation catalysed by sesquiterpene synthases to form carbocation intermediates from which >300 different terpene skeletons can form.

When engineering terpene synthases the high reactivity of the carbocation intermediates along the reaction trajectory allows for manipulation of the reaction to produce alternative final products. Subtle changes in the enzyme active site by mutagenesis frequently alter product distribution. We and others have repeatedly shown that simple point mutations can be used to engineer variants with different product distributions.^41,52–66^ It should be noted that not all these terpene synthase variants have equivalent catalytic efficiency as the WT enzyme.^67–69^ For C15 farnesyl pyrophosphate >300 different hydrocarbon skeletons are possible (Fig. 2).^52^ Predictively engineering terpene synthases and exploiting screening methods for selection of new activities must overcome challenges with controlling the enzyme activity and product distribution to obtain enzymes that both produce the desired products and are catalytically efficient.

### 2.3. Nucleophilic water capture or deprotonation of final carbocation

Quenching of the final carbocation within terpene synthase reaction cascades involves either direct deprotonation to produce hydrocarbon products or nucleophilic attack by water to generate oxygenated terpenes. Crystal structures of class I and II terpene synthases surprisingly show that water molecules are often held in close proximity to the product or substrate analogues within the active site even for enzymes that do not generate hydroxylated products.^70–73^ Understanding how terpene synthases control the exclusion or addition of water to precise carbocationic intermediates and at specific sites with defined stereochemistry is important for engineering terpene synthases. Generating hydroxylated terpenoids is of particular interest as regio- and stereospecific hydroxylation of non-activated hydrocarbons is chemically challenging and often requires specialised enzymes such as cytochrome P450s.

Originally, by using isotope labelled water, H_2_^18^O, it was unambiguously established for several terpene synthases that hydroxylation occurs through quenching with water derived from bulk solvent.^74–76^ This discriminated from an alternative mechanism where recapture of the anionic pyrophosphate is followed by hydrolysis (*e.g.*, bornyl pyrophosphate synthase).^77^ Although it is recognised that the nucleophilic water for terpene synthases is derived from bulk solvent it is also essential in catalysis that bulk water is excluded from the active site to avoid unproductive premature quenching of the carbocationic intermediates. For class I enzymes, binding of pyrophosphate of the substrate in the metal binding pocket leads to closure of the active site when forming the substrate bound Michaelis complex.^17,78–80^ This closed species is formed immediately prior to catalysis and carbocation formation and likely excludes bulk water from the active site.

### 2.4. Role of active site water in germacradien-4-ol and aristolochene synthases

**Fig. 3:**
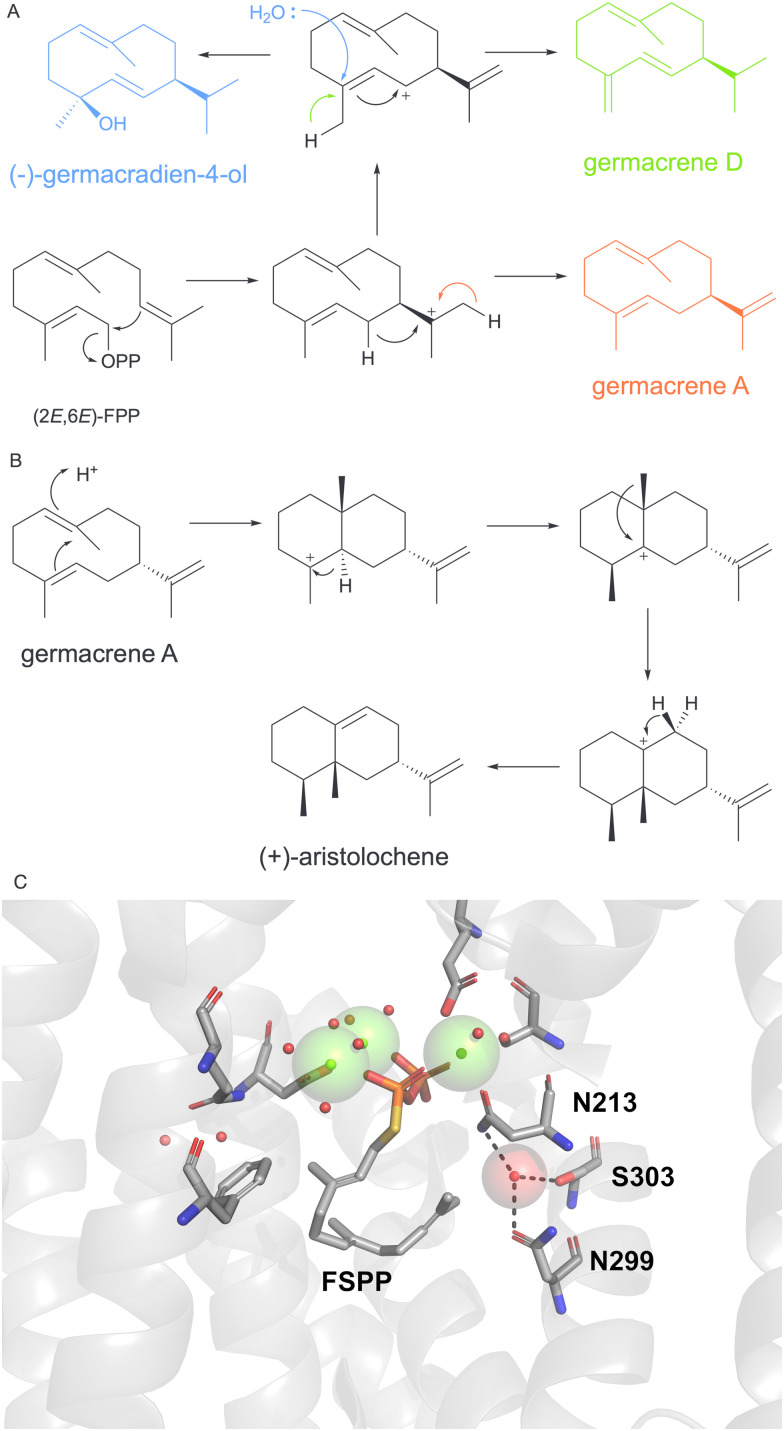
Role of active-site water in Gd4olS and AT-AS. (A) Proposed cyclisation mechanism of FPP to (−)-germacradien-4-ol and alternative products by Gd4olS. (B) Proposed mechanisms of AT-AS formation of (+)-aristolochene from neutral intermediate germacrene A. (C) AT-AS active site structure (PDB: 4KUX). Active site water (red sphere) is located adjacent to substrate analogue and forms hydrogen bonds (black dotted lines) to K and H helices. Magnesium ions are depicted by green spheres.

Germacradien-4-ol synthase (Gd4olS) from *Streptomyces citricolor* catalyses the 1–10 cyclisation of (2*E*,6*E*)-FPP to the hydroxylated product (−)-germacradien-4-ol (Fig. 3A).^81^ After first confirming that the final nucleophilic attack occurs from water derived from bulk solvent, we investigated the role of active site water.^74^ Mechanistically, Gd4olS likely catalyses a 1,3-hydride shift from germacrenyl cation to yield the final allylic cation, which is quenched on C3 by water. In collaboration with Christianson, we successfully obtained an X-ray crystal structure of Gd4olS in the open conformation. As for other terpene synthases crystallised water was positioned in the active site. The active site lacked any obvious polar residues which could act as the general base. The pyrophosphate itself is often proposed as a suitable base for catalysis. Comparison to closed structures of *Aspergillus terreus* aristolochene synthase (AT-AS) with the substrate analogue farnesyl thiolodiphosphate (Fig. 3C, PDB: 4KUX) showed that for AT-AS a conserved water molecule coordinates to the asparagine of the NSE (N213) motif and the sidechains of N299, S303 of the K helix in open and closed structures.^71^ The water molecule is in van der Waals contact with the C12/C13 positions of the substrate analogue. For Gd4olS, the equivalent H and K helix residues comprise N218, Y303 and E307. Mutation of the NSE asparagine 218 to glutamine reduced quenching with water and produced ∼50% germacrene A and trace quantities of germacrene D. As the metal binding capacity of N218 relies on the carboxyl group other variants were inactive or displayed significantly compromised activities. Variants of Y303 and E307 on the K helix and which neighbour N218 also compromised catalysis indicating a role in carbocation stabilisation, substrate folding and potentially water management.

For AT-AS which catalyses the production of (+)-aristolochene (Fig. 3B), the triad of K and H helix residues (N213, N299 and S303) were similarly scrutinised by site-directed mutagenesis.^82^ Unlike Gd4olS, AT-AS must prevent nucleophilic attack by water even though the water is positioned adjacent to the substrate. All variants compromised cyclisation and generated greater proportions of the neutral intermediate germacrene A as well as linear hydroxylated products nerolidiol and farnesol. Water management of the active site may therefore control not only the nucleophilicity of active site water but also the folding of the substrate and steric control over the reaction trajectory. AT-AS exhibited for the first time that water molecules can be catalytically inert and form part of the active site contour and assist substrate folding. The nerolidiol produced from AT-AS variants forms as a racemic mixture indicating that any control over water capture is missing and possibly that bulk water has access to the active site. In the open structure of Gd4olS, the adjacent H-1α and F-G loops were disordered. The K and H helices may therefore play a role in exposure to bulk water though the dynamics of the active site loops that are involved in closure with substrate binding.

**Fig. 4:**
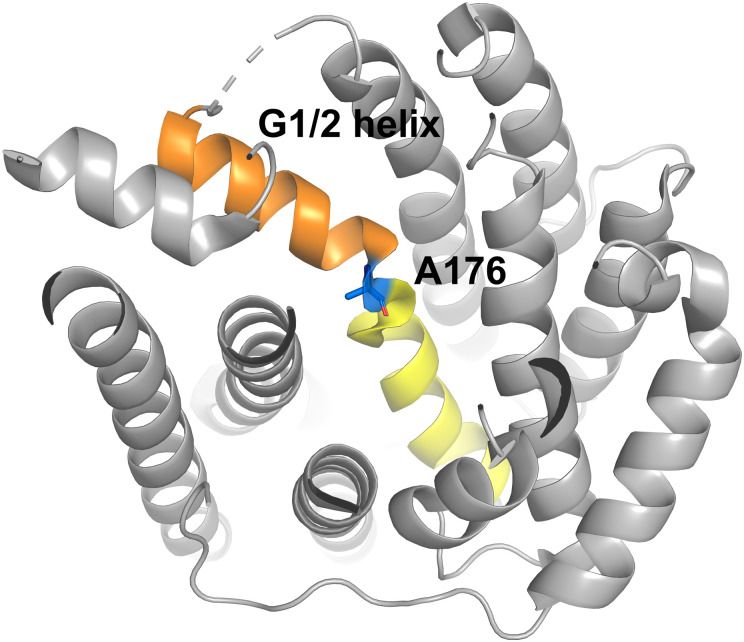
Structure of Gd4olS (PDB: 5I1U) with G1/2 helix highlighted in orange and yellow around residue A176 (blue) at the helix ‘kink’.

More recently, we investigated the role of the G1/2 helix break motif of Gd4olS in water capture. The G helix ‘kink’ which splits the helix into two is crucial for folding of the substrate in the active site and known to strongly alter product distributions (Fig. 4).^83^ For *Streptomyces clavuligerus*, 1,8-cineole monoterpene synthase replacement of a single asparagine residue in the G1/2 helix break with aliphatic residues was sufficient to abolish water capture and produce non-hydroxylated products by displacing an adjacent water molecule.^83^ In Gd4olS, variants of A176 with large polar and nonpolar residues were created to similarly displace any neighbouring water at the G1/2 helix ‘kink’.^53^ The polar variants continued to produce germacradien-4-ol suggesting that direct coordination of water for Gd4olS at this site is unlikely. Exchange of A176 to nonpolar residues however led to accumulation of germacrene A and germacrene D. The largest accommodated change, A176M produced 90% germacrene A. We anticipated that in this case the larger aliphatic groups disrupt the geometry of the active site including the π-carbocation interactions provided by aromatic residues and favour premature deprotonation to produce germacrene A.

### 2.5. δ-Cadinene synthase active site volume balances δ-cadinene and germacradien-4-ol product distribution

**Fig. 5:**
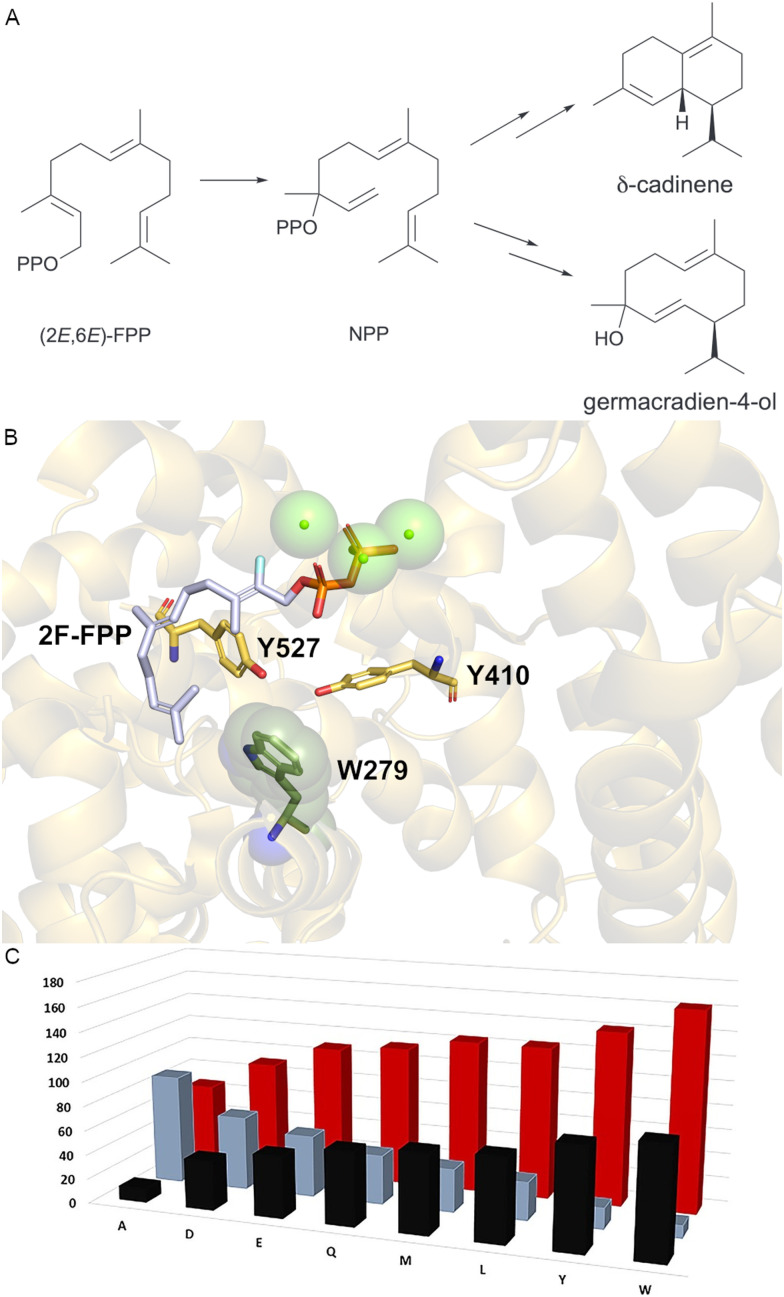
Engineering DCS water capture. (A) DCS produces δ-cadinene and minor product germacradien-4-ol *via* nerolidyl pyrophosphate (NPP). (B) DCS active site structure (PDB: 3G4F). W279 is depicted as dark green spheres, Magnesium ions as green spheres and 2-fluoroFPP as sticks. (C) Histogram of percentage δ-cadinene (black) and germacradien-4-ol (grey) for variants of W279, alongside van der Waals volume (red). Part C of this figure was reproduced from ref. 66 under the CC BY 4.0 licence.

High-fidelity terpene cyclase δ-cadinene synthase (DCS) from *Gossypium arboreum* catalyses a similar cyclisation to Gd4olS but instead of final nucleophilic attack by water it deprotonates from the C6 position to produce δ-cadinene (Fig. 5A).^84,85^ DCS must therefore exclude nucleophilic water. The native enzyme produces a small quantity (∼2%) of germacradien-4-ol alongside the major product δ-cadinene (98%). Although catalysing similar mechanisms, Gd4olS and DCS are not related and show low sequence identity. Unlike Gd4olS, DCS lacks the NSE motif and rather has additional aspartate residues to coordinate the catalytic magnesium. Keasling *et al.* screened variants of DCS using medium throughput methodology and identified 21 variants where germacradien-4-ol levels increased.^86^ The altered amino acids were distributed around the G1/2 helix within the active site. From these variants, saturation mutagenesis was used to establish the improved variant N403P/L405H which produced 53% germacradien-4-ol *in vitro*.

While probing the role of the β-domain in DCS, we similarly showed that N-terminal truncation can alter solvent accessibility and lead to larger quantities of germacradien-4-ol.^87^ We were successful in obtaining X-ray crystal structures with and without substrate analogues (Fig. 5B).^73^ No water molecules were in the site adjacent to K or H helices previously observed to contain structurally conserved water in AT-AS. We generated variants of W279 within the active site and identified a clear correlation between the van der Waals volume of the residue and the distribution between germacradien-4-ol and δ-cadinene (Fig. 5C).^66^ Smaller volume residues resulted in greater nucleophilic attack by water. The single amino acid change W279A resulted in ∼90% alcohol product. In the crystal structure, W279 is directly adjacent to Y527 and Y410 at the base of the active site. Y527 is positioned on the K helix in place of the water bound in AT-AS substrate analogue complexes. By increasing the volume of the active site, water likely enters which in turn increases the propensity for nucleophilic attack and germacradien-4-ol production.

### 2.6. Water capture of class I diterpene synthases

**Fig. 6:**
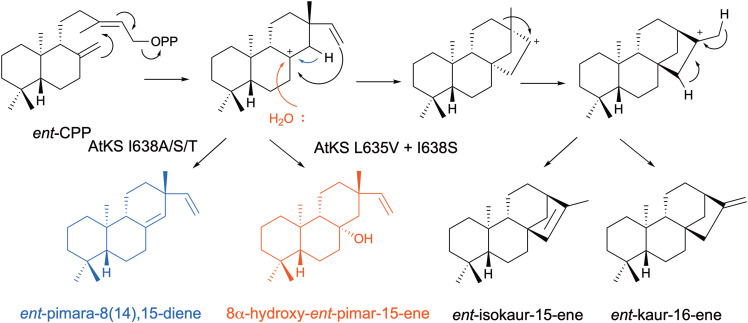
Mechanism of kaurene-like synthase cyclisation *via* initial ionisation of *ent*-CPP. AtKS variants were engineered to produce hydroxylated (red) and non-hydroxylated (blue) diterpene products.

Kaurene-like synthases (KSL) catalyse the class I cyclisation step for labdane related diterpenoid biosynthesis from copalyl- (CPP) and *ent*-copalyl pyrophosphate (*ent*-CPP) which are first formed from GGPP by class II synthases.^88^ Depending on the terpene synthase, different mechanisms are employed resulting in either deprotonation or quenching with water from diverse final carbocations. By comparing different related KSL enzymes Peters *et al.* identified a conserved isoleucine within the G1/2 helix ‘kink’, which when mutated altered the product distribution.^89,90^ For *Oryza sativa* KSL5i, the I664T variant altered cyclisation (*ent*-isokaur-15-ene/*ent*-pimara-8,15-diene switch) but did not result in water capture.^89^ For *Arabidopsis thaliana* KS the I638A/S/T variants similarly favoured *ent*-pimara-8(14),15-diene formation but also produced the minor hydroxylated product, 8α-hydroxy-ent-pimar-15-ene (Fig. 6).^90^ By combining isoleucine variants with an additional synergistic site on the G1/2 helix (AtKS L635V) the diterpene alcohol was made as the major product.^91^

**Fig. 7:**
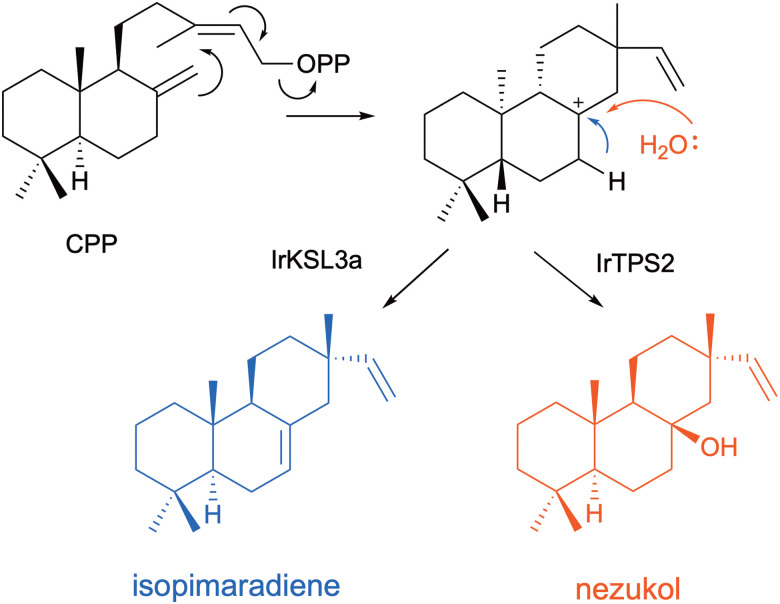
Cyclisation of CPP to non-hydroxylated and hydroxylated diterpenes by two KSL enzymes from *Isodon rubescens* which share high sequence identity.

In a similar approach, two KSL terpene synthases from *Isodon rubescens* (IrKSL3a and IrTPS2), which share 98% sequence identity were compared. IrKSL3a produces isopimaradiene, while IrTPS2 produces hydroxylated nezukol (Fig. 7).^92^ Switching residue A523 of IrTPS2 to isoleucine as found for IrKSL3a abolished hydroxylation.^93^ Molecular dynamics (MD) simulations of the enzyme model identified regions close to the C8 position of CPP where water is likely to position between residues S499 and T527. A523 sidechain neighbours the trapped water molecule. When A523 was exchanged to isoleucine water capture was prevented, most likely because the β-methyl of the isoleucine sidechain displaced the water molecule positioned between the serine/threonine dyad. Leucine and residues with smaller volume sidechains were unable to avoid hydroxylation. Engineering with the opposite aim to introduce hydroxylation for IrKSL3a required further complexity. The equivalent reverse mutation I522A in IrKSL3a alone and in combination with A498S which introduced the water coordinating serine of IrTPS2 produced only small quantities of nezukol. To selectively produce nezukol the variant A498S/I522A/E632K/L523I was necessary.

### 2.7. Simulation-guided engineering of terpene synthase water capture

The fact that active site residues roles are interconnected makes it challenging to separate mechanisms which drive quenching of the carbocation with water from mechanisms of cyclisation. When engineering new enzyme variants to introduce or avoid hydroxylation by shifting the balance between quenching with water or deprotonation linear hydrocarbon and/or hydroxylated products need to be avoided. For these products, such as farnesol, farnesene, linalool, nerolidiol, the catalytic cycle is significantly impeded and usually indicates that the substrate is unable to fold appropriately within the active site. In collaboration with Marc van der Kamp at the University of Bristol we have recently demonstrated a strategy using MD simulations to identify mutations that lead to acyclic products and can therefore be discarded prior to experimental analysis and to recognise regions where active site water is present and can be targeted for engineering.

**Fig. 8:**
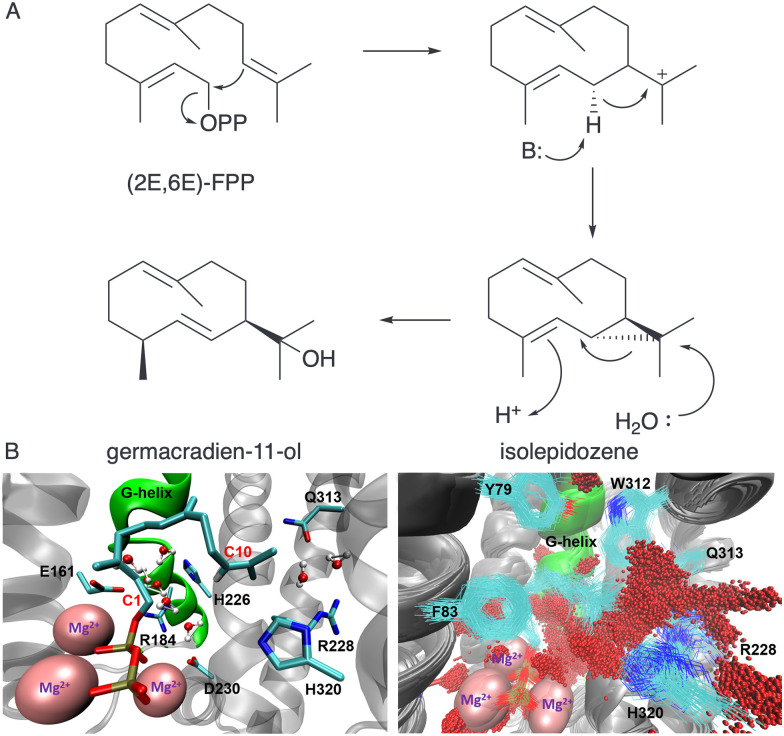
Germacradien-11-ol synthase catalysis. (A) Proposed mechanism *via* neutral isolepidozene intermediate. (B) Structure of Gd11ol active site (left pane) highlighting G-helix and RQH sites. Superimposed MD structures (right pane) illustrating the location water molecules (red spheres). This figure is reproduced as a modified version from ref. 55 under the CC BY 4.0 licence.

We first established the method to reduce water capture for germacradien-11-ol synthase (Gd11olS) from *Streptomyces coelicolor* which forms a hydroxylated product in the biosynthesis of geosmin (Fig. 8A).^55,94^ During MD simulations of 30 ns the C1–C10 distance was monitored for different substrate binding modes and the likely productive binding conformation of FPP that results in the germacrenyl cation, established. Two regions involved in water interactions were observed in simulations, namely the ‘RQH’ site comprising R228, Q313 and H320 (adjacent to FPP:C11) which is equivalent to the previous conserved water site for AT-AS (N299, S303 and V212) and the G1/2 helix ‘kink’ (Fig. 8B). Water was shown to ‘flow’ between these sites, although only the G helix site is exposed to bulk water on the timescale of the simulations. Variants were designed to target water management at both sites. Reduced polarity at the RQH site resulted in minor production of non-hydroxylated products, typically germacrenes whereas mutation of the G helix ‘kink’ region had a more significant impact on product distribution. For G188S and A190V, the non-hydroxylated isolepidozene was produced alongside germacradien-11-ol, germacrene D and germacrene A. For the G188A variant isolepidozene was produced as the major product. Isolepidozene is proposed as the neutral intermediate of the reaction. In MD simulations, the RQH site appeared to hold water proximal to C11 of FPP for nucleophilic attack (Fig. 8B). Engineering of the G-helix likely alters water binding at this site.

**Fig. 9:**
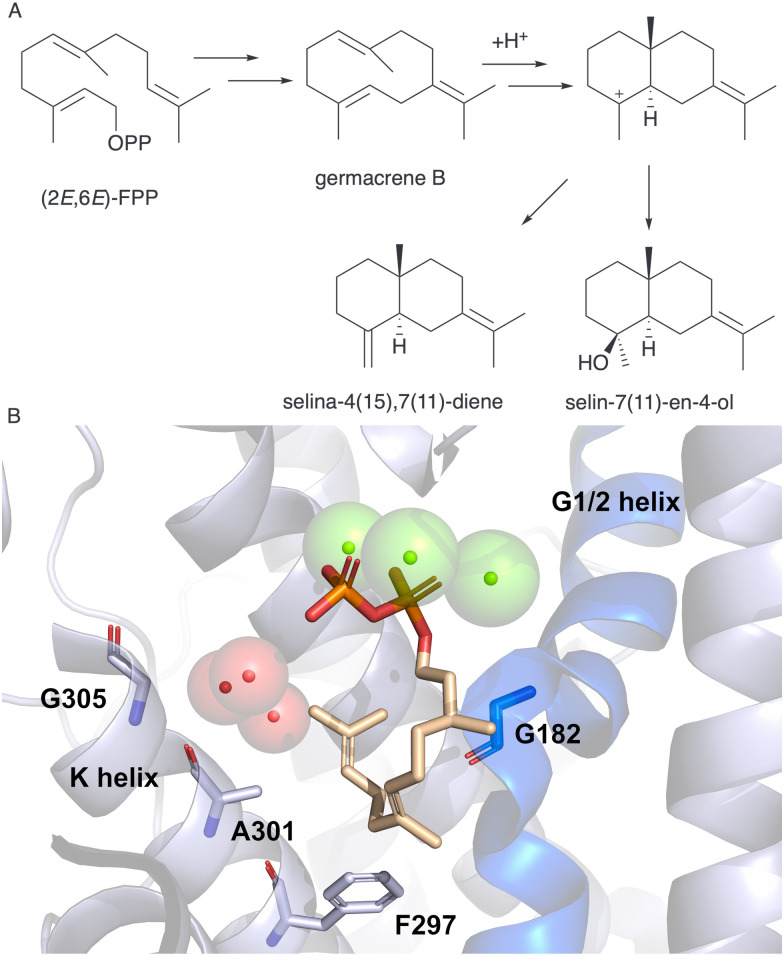
(A) Abbreviated scheme of the proposed mechanism of SdS production of selina-4(15),7(11)-diene and alternative hydroxylated product selin-7(11)-en-4-ol. (B) SdS active site structure (PDB: 4OKZ), G1/2 helix depicted in blue and magnesium ions as green spheres. Active site water adjacent to K and H helices shown as red spheres. Substrate analogue dihydroFPP is displayed as sticks.

A MD simulation-led mutation strategy was next employed to introduce hydroxylation for selenadiene synthase (SdS) from *Streptomyces pristinaespiralis* ATCC 25486 which produces selina-4(15),7(11)-diene (Fig. 9A).^95,96^ The crystal structure of SdS bound to a substrate analogue showed three water molecules located between the H- and K-helices (Fig. 9B).^17^ Again, hydroxylation is not catalysed even though water is in the active site. Dickschat *et al.* propose from QM/MM simulations that active site water plays a role in the deprotonation and reprotonation sequence of the neutral intermediate germacrene B and acts as a bridge to the general base/acid backbone carbonyl of Gly182.^97^ By following the C1–C10 distance during our MD simulation we were able to establish variants in the K helix where cyclisation would not be significantly compromised to probe the role of the water molecules adjacent to the K-H helix site.^96^ Only F297A was anticipated to impact cyclisation and was subsequently shown experimentally to produce acyclic β-farnesene alongside selena-4(15),7(11)-diene. Variants of A301 on the K helix mostly led to germacrene B without impacting hydroxylation. The G305E variant in the K helix introduced hydroxylation and produced ∼20% selin-7(11)-en-4-ol (Fig. 9A). QM/MM simulations showed that for the G305E variant, water approaches the C3 carbocation consistent with producing the *S* configuration in the product and which was not observed for WT SdS. The pyrophosphate is suitably placed to act as general base. By manipulating the pH of the reaction, the proportion of selin-7(11)-en-4-ol could be further increased. By this strategy we established the first known enzymatic formation of selin-7(11)-en-4-ol.

### 2.8. Active site loop engineering to abolish water capture

Patchoulol synthase (PTS) from *Pogostemon cablin* catalyses the formation of hydroxylated fragrance patchoulol.^98^ MD simulations were used to establish substrate binding mode by monitoring the C1–C10 distance and capacity to form the *R*-germacryl cation.^99^ Again, simulations were useful for identifying key residues involved in substrate folding that, when mutated, result in acyclic products. A water molecule was identified to likely position adjacent to residue Y525 and C6 of FPP. Mutation of this site, (Y525F) resulted in major products β-caryophyllene (40%) and α-bulnesene (30%). The production of β-caryophyllene indicated that Y525 influences not only hydroxylation but balances the relative stability of C1–C11 and C1–C10 cyclisation. Y525A likewise abolished hydroxylation but had different distributions between the C1–C10 and C1–C11 products. Similarly, mutation of the G1/2 helix ‘kink’ (C405A) as for other terpene synthases was effective at producing non-hydroxylated products.

**Fig. 10:**
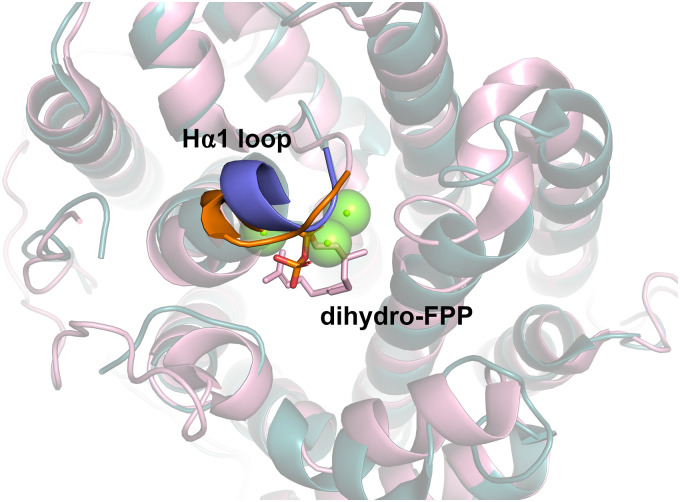
Aligned structures of Gd11olS (blue, PDB: 5DZ2) and SdS (pink, PDB: 4OKZ). Hα1 loop from Gd11olS highlighted in purple and SdS in orange. Chimeric enzymes where Ha1 loops were exchanged was effective for altering product distribution and avoiding water capture.

For an alternative and more general strategy to engineer water capture we also probed the role of the active site Hα-1 loop.^99^ In the open-to-closed transition this loop undergoes substantial conformational change. Designing chimeras between closely related enzymes can be effective for identifying key residues involved in water capture.^100^ For both Gd11olS and PTS, we exchanged four amino acids of the Hα-1 loop with the equivalent residues from the non-hydroxylating SdS (Fig. 10). In both cases, the chimeras avoided hydroxylation and resulted in accumulation of neutral intermediates from the reaction coordinate, *i.e.*, isolepidozene (Gd11olS) and germacrene A and α-bulnesene (PTS).

## 3. Exploiting non-natural substrate analogues

Terpene substrate analogues have been indispensable to investigate the catalytic mechanisms of terpene synthase. Unreactive farnesyl-*S*-thiolodiphosphate is commonly used in X-ray crystallography of sesquiterpene synthases for ground-state enzyme-substrate complexes.^71,101,102^ Fluorinated substrate analogues have been used to support cyclisation mechanism as fluoro substituents have an electronic effect and can alter carbocation stabilities. Likewise, isotopic labelling has been crucial for following cyclisation reaction mechanisms as well as methyl and hydride shifts.^103^

### 3.1. Fluorinated substrates

**Fig. 11:**
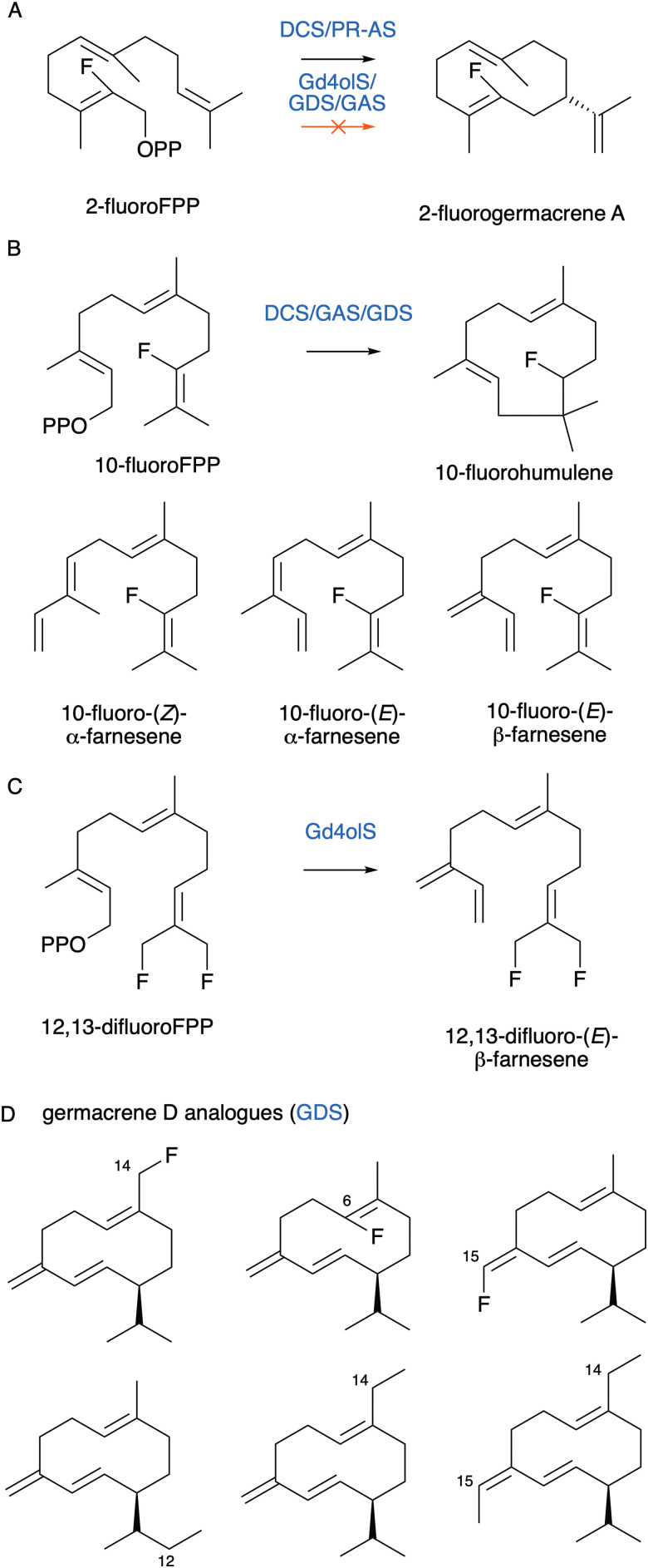
Fluorine substituted sesquiterpene products. (A) 2-FluoroFPP is a substrate for DCS and PR-AS but not Gd4olS, GDS or GAS. (B) 10-FluoroFPP favours a C1–C11 cyclisation to produce 10-fluorohumulene as well as farnesene analogues. (C) Fluorine substituted farnesene produced by Gd4olS. (D) Germacrene D analogues produced from FPP analogues.

Terpene synthase promiscuity means that substrate analogues frequently turnover to produce novel products. For instance, 2-fluoro-FPP and 10-fluoro-FPP have been used to explore DCS cyclisation mechanism but also act as substrates and produce 2-fluorogermacrenes, 10-fluorohumulene and fluoroinated farnesenes (Fig. 11).^84^ Similarly, 2-fluoro-FPP is a substrate for *Penicillium roqueforti* aristolochene synthase (PR-AS), which converts it to 2-fluorogermacrene A (Fig. 11A).^104^ 2-Fluoro-NPP is a substrate for limonene synthase from *Citrus sinensis.*^105^ In contrast, 2-fluoro-FPP is an unreactive inhibitor of Gd4olS.^74^ This suggests that initial ionisation and cyclisation mechanisms differ between terpene synthases. Where products form with 2-fluoroFPP, ionisation and cyclisation are likely concerted while where it acts as an inhibitor, like in Gd4olS catalysis, initial ionisation to the farnesyl cation likely occurs prior to cyclisation. As such turnover of fluorinated substrates to novel products is enzyme dependent. For Gd4olS, the alternative 12,13-difluoro-FPP was turned over to produce 12,13-difluoro-(*E*)-β-farnesene (Fig. 11C). Further examples of halogenated terpenes include 7-fluoroverticillenes^106^ and 1-fluorogermacrene A.^107^

### 3.2. Germacrene A and D analogues

We investigated fluorinated and methylated analogues with germacrene A synthase (GAS) and germacrene D synthase (GDS) from *Solidago canadensis*, both of which undergo a 1–10 cyclisation to germacryl cation.^67,108,109^ Substituents were placed at different sites of the FPP substrate and incubated with each enzyme. 2-Fluoro-FPP and 15-methyl-FPP were not substrates of GAS or GDS. The 6-fluoro-, 14-fluoro and 14-methyl-substituted FPP were turned over but did not alter cyclisation and produced fluorinated/methylated germacrene A and D analogues (Fig. 11D). 15-Fluoro-FPP and 12-methyl-FPP were likewise substrates for GDS but not GAS and produced substituted germacrene D. The 10-fluoro-FPP analogue altered cyclisation through electron-withdrawing effects of the substitution and both enzymes produced α-10-fluoro-humelene. For GDS, 10-fluoro-(*E*)-β-farnesene was also made. Active site engineering of residues Y524, W275 and Y406 was used to expand the substrate scope of GDS further. Y406F improved the efficiency of the enzyme for the native substrate, FPP. This variant was also able to produce (*S*)-15-methylgermacrene D and (*S*)-14,15-dimethylgermacrene D from 15-methylFPP and 14,15-meFPP originally poor substrates for the WT enzyme (Fig. 11D). Effects of these new analogues on the grain aphid *Sitobion avenae* were investigated using GC-EAG and behavioural bioassays and showed semiochemical responses for the analogues as well as the natural (*S*)-germacrene D which acts as a repellent.^67^ Interestingly, (*S*)-14,15-dimethylgermacrene D had reversed the repellent activity and acted as an attractant with application for insect pest management.

### 3.3. Substrates analogues

**Fig. 12:**
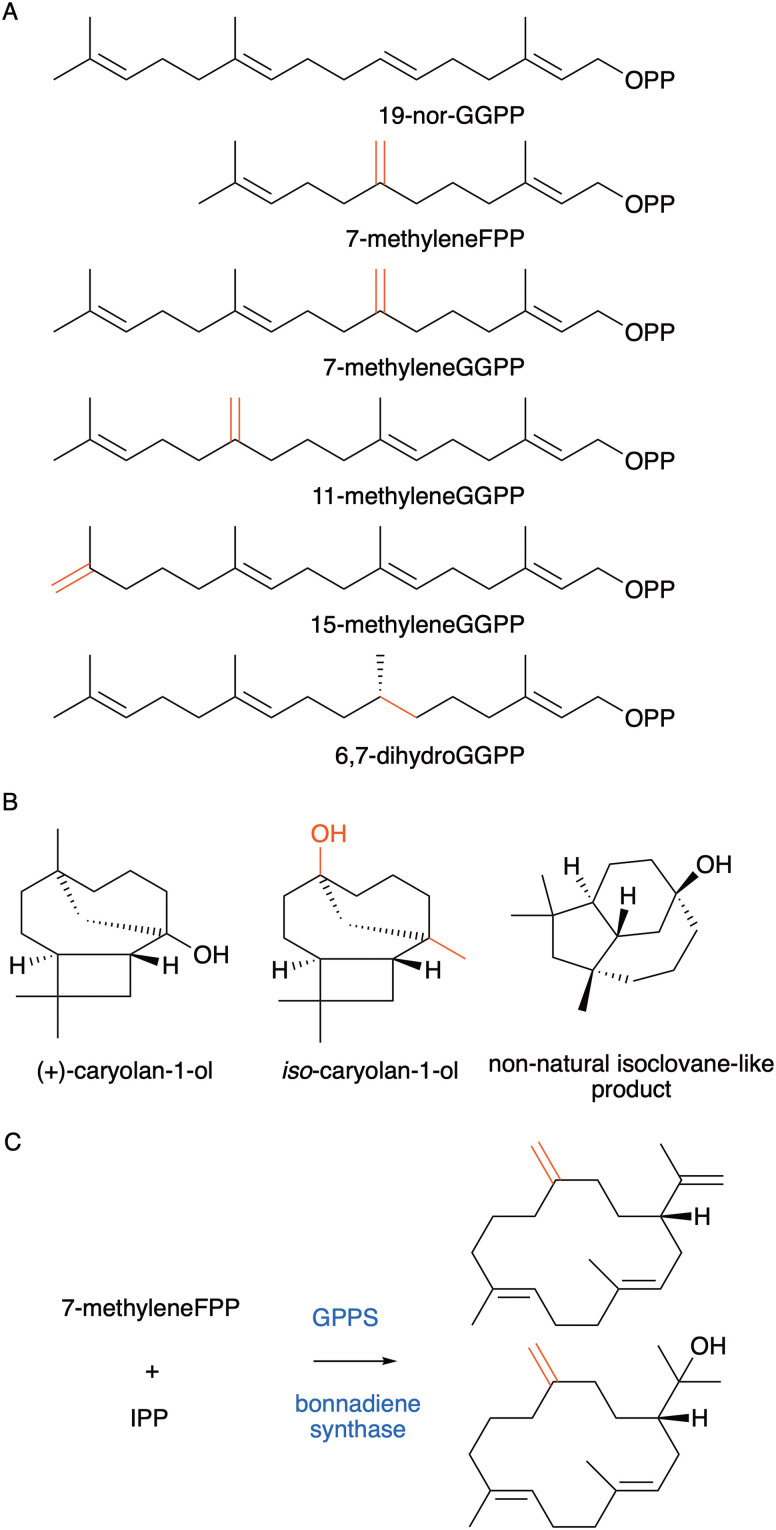
Non-natural terpene synthase substrates and products with (A) altered number of carbon atoms, double bonds, and double bond positions. (B) Natural (+)-caryolan-1-ol produced from (2*E*,6*E*)-FPP and non-natural iso-caryolan-1-ol from 7-methyleneFPP. Novel isoclovane-like product produced from 7-methyleneFPP. (C) Casbene analogues with altered double bond positions produced from substrate analogues.

In addition to increasing the number of carbon atoms, the removal of methyl groups from the substrate has also been successful. 19-Nor-GGPP (Fig. 12A) was incubated with twenty different diterpene synthases and twenty-five different novel C19 products were obtained.^110^ For certain enzymes, cyclisation was unaffected and equivalent 19-nor analogues of the natural products were formed. For others, alternative cyclisation products were observed due to the impact on the carbocation stabilities and substrate binding conformations. It is similarly possible to shift double bonds of prenyl pyrophosphate substrates. For instance, 7-methyleneFPP was converted to the natural product aristolochene by PR-AS (Fig. 12A).^111^ Incubation of the same substrate with tricyclic caryolan-1-ol synthase from *Streptomyces griseus* led to a new isomer of the natural product, iso-caryolan-1-ol (Fig. 12B), where the alcohol and methyl groups had exchanged position.^112^ Incubation with presilphiperfolan-8-β-ol synthase (BOT2) from *Botrytis cinerea* produced a novel hydroxylated product with new isoclovane-like skeleton not previously found in nature (Fig. 12B).^112^ Likewise, by shifting the C6–C7 double bond to C7–C8 a new germacrene derivative was formed with protoillude and viridiflorene synthases.^113^ Novel germacrene analogues were also produced by shifting methyl groups of prenyl pyrophosphate substrates.^114^ Extension of 7-methyleneFPP to isogeranylgeranyl pyrophosphate with IPP enabled the synthesis of casbene analogues with exocyclic double bonds (Fig. 12C)^115^ and further novel compounds with unique skeletons were made from 7-methyleneGGPP, 11-methyleneGGPP and 15-methyleneGGPP.^116^ By shifting C10–C11 double bond to the C11–C12 position of 18-nor-GGPP and GGPP two new 12-membered cyclic terpene products were produced with spata-13,17-diene synthase (SpS) from *Streptomyces xinghaiensis*.^117^

For enzymes where the C6–C7 double bond directly participates in the reaction, the C6–C7 double bond of GGPP was saturated to interrupt the cyclisation mechanism of diterpene synthases.^118^ Novel products with unique terpene skeletons were produced. For enzymes where the double bond does not directly participate in cyclisation, products from the same substrate were either saturated equivalents of the natural enzymatic product or showed changes in cyclisation most likely due to conformational changes in the substrate because of the increased degrees of freedoms available at the saturated site.

### 3.4. Oxygenated substrate analogues

**Fig. 13:**
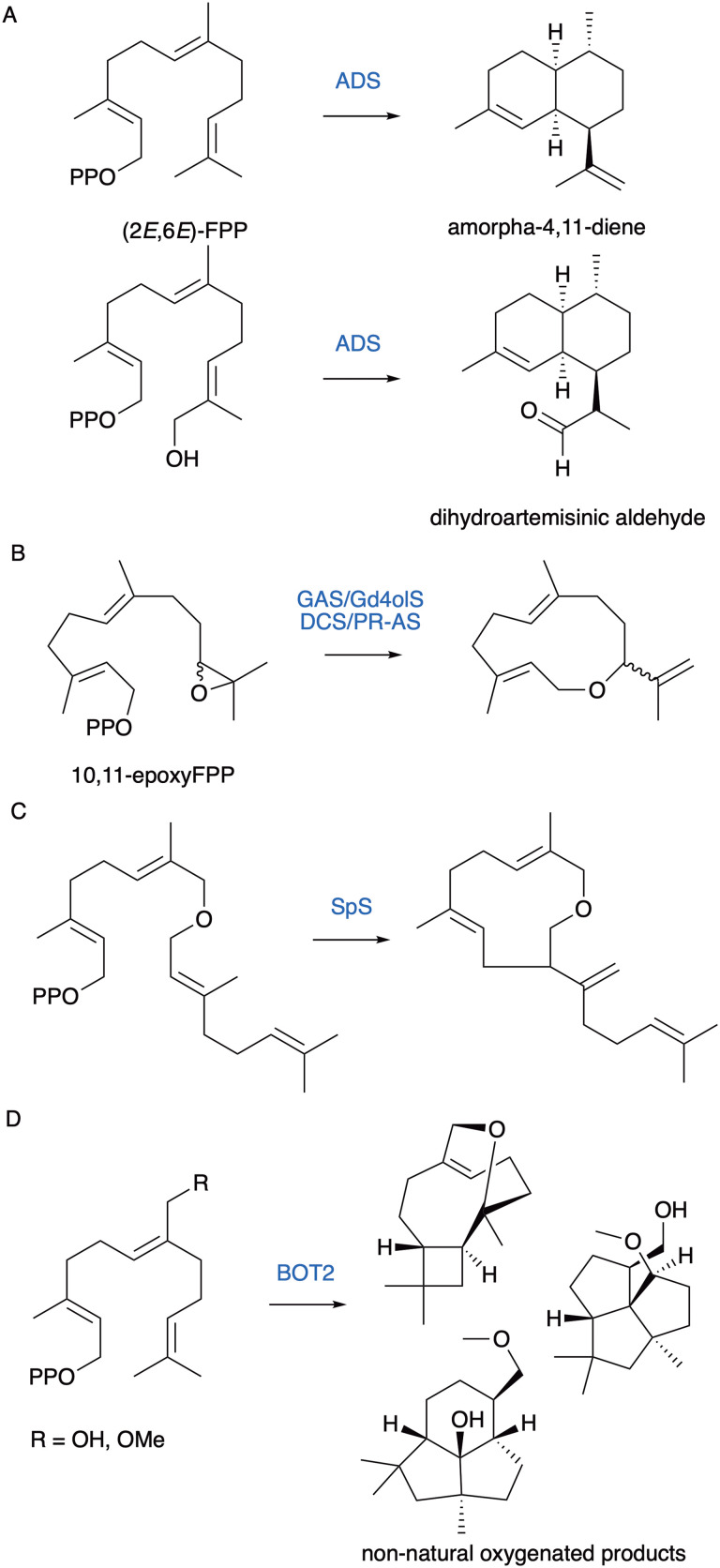
Examples of non-natural oxygenated terpenes synthesised from prenyl pyrophosphate analogues. (A) ADS catalyses the cyclisation of FPP to amorpha-4,11-diene. ADS produced dihydroartemisinic aldehyde from 12-hydroxyFPP. (B) Several terpene synthases catalyse C1–C10 cyclisation of 10,11-epoxyFPP to produce a novel macrocyclic ether. (C) Prenyl pyrophosphate analogue with main chain ether produced novel 11 membered ether product. (D) 14-Hydroxy- and 14-methoxy-FPP are substrates of BOT2 and produced three tricyclic products as major products.

We further explored the substrate scope of terpene synthases with oxygenated substrates. By using the substrate analogue 12-hydroxyFPP, as well as 12- and 13-acetoxyFPP we were able to exploit the relaxed substrate scope of amorphadiene synthase (ADS) to produce (11*R*)- and (11*S*)- dihydroartemisinic aldehyde (Fig. 13A).^119^ The aldehyde is an advanced precursor for the synthesis of antimalarial artemisinin that would otherwise require multiple redox steps including two cytochrome P450 oxidations of amorphadiene for synthesis from the natural FPP substrate. The synthesis to artemisinin was demonstrated from substrate analogues on a small scale and produced two epimers.^120^ In addition to the natural compound the unnatural epimer activity is still to be investigated and may be useful with other analogues of artemisinin for overcoming the increasing problem of resistance.

10,11-EpoxideFPP and a related allylic alcohol (10-hydroxy-11-methyleneFFP) containing FPP analogue were tested with the C1–C10 cyclisation enzymes Gd4olS, GAS, DCS and PR-AS.^121^ For all, germacryl cation formation was diverted through the intramolecular nucleophilic oxygen capturing the initial carbocation and formed a novel macrocyclic ether (Fig. 13B). FPP analogues with unnatural ether and thioether linkages within the main chain have also been explored with several sesquiterpene synthases.^122–125^ Cyclisation is often but not exclusively retained, and the heteroatom incorporated to generate cyclic and acyclic ether and thioether derivatives. Extension of a FPP ether to a GGPP analogue produced a novel terpene with heteroatom incorporation with SpS (Fig. 13C).^117^ 14-hydroxyFPP and 14-methoxyFPP were likewise converted by BOT2 into three tricyclic novel products, one of which is formed by migration of the methoxy group *via* a proposed 1,4-Wagner Meerwein rearrangement (Fig. 13D).^126^

### 3.5. Exploiting DMAPP and IPP analogues

**Fig. 14:**
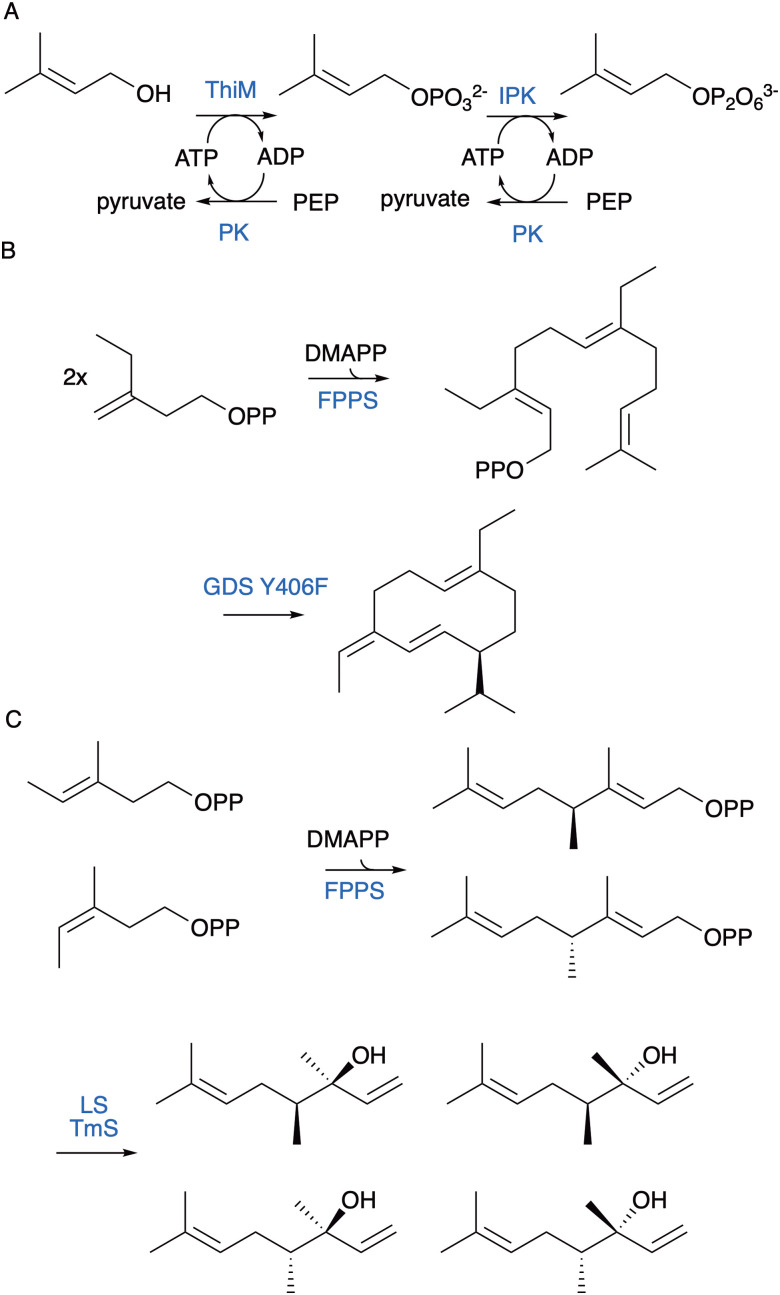
Chemoenzymatic synthesis of terpenes from prenols pyrophosphate building blocks. (A) Enzymatic phosphorylation of prenol to DMAPP. (B) Methyl isoprenyl pyrophosphate were used with a farnesyl pyrophosphate synthase and GDS Y406F terpene synthase to produce 14,15-dimethylgermacrene D. (C) (*Z*)- and (*E*)-4-methyl-IPP were used to synthesise C11 linalool analogues.

More recently, assembly of terpene substrate analogues from DMAPP and IPP analogues has been developed. We established a new chemoenzymatic route using promiscuous kinases to pyrophosphorylate prenol, isoprenol and their analogues (Fig. 14A).^127,128^ A range of kinases have now been demonstrated to phosphorylate prenol and isoprenol including hydroxyethylthiazole kinase,^127,129^ choline kinase,^130^ diacylglycerol kinase^129^ and acid phosphatase PhoN.^131^ Isopentenyl monophosphate kinase (IPK) from the ‘archaeal’ mevalonate pathway, which bypasses mevalonate-5-phosphate is used for the second phosphorylation step.^132–134^ Coupling enzymes to ATP recycling generates quantitative yields. Intermediates can then be assembled by PTs into GPP, FPP and GGPP and their analogues with additional alkyl- and hydroxyl-groups with yields of up to 80%.^127^ The strategy was a significant improvement on the chemical synthesis of 14,15-dimethylFPP and production of the non-natural homoterpene aphid attractant 14,15,dimethyl-germacrene D (Fig. 14B). It also enabled the introduction of heteroatoms, and we were able to produce dihydroartemisinic aldehyde from 4-hydroxyprenol *via* 12-hydroxyFPP. Similar approaches with chemical phosphorylation of C4-6 alcohol precursors have been successful for introducing methyl substituents. For instance, 2-methylisoborneol was produced using a non-natural pathway from (*S*)- and (*R*)-2-methyl-IPP.^135^ Similarly, analogues of variediene with new skeletons and methyl substitutions were made from five methylated DMAPP analogues.^136^ (*Z*)- and (*E*)-4-methyl-IPP were likewise incorporated into homoGPP and homoFPP for the final extension-step prior to conversion to 4-methyllinalools and methylated T-muurolol analogues (Fig. 14C).^137^

### 3.6. Naturally produced and engineered non-canonical homoterpenes

**Fig. 15:**
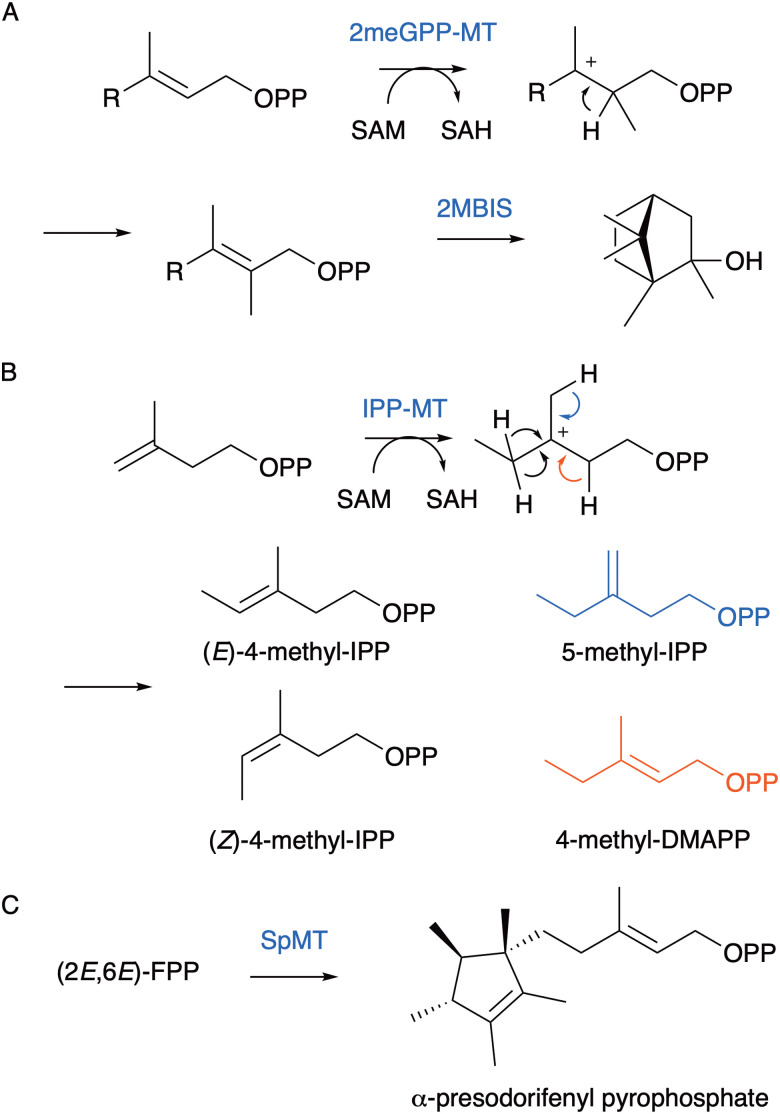
Methyltransferases produce terpenes with non-canonical numbers of carbon atoms. (A) 2-Methyl-GPP is produced by SAM-dependent methylation. 2-Methylisoborneol synthase (2MBIS) catalyses cyclisation to non-canonical C11 product. (B) Examples of IPP and DMAPP analogues that can be produced using methyltransferases and extended to novel terpene products. (C) Unusual methyltransferase from *S. plymuthica* installs a methyl group and cyclises product to αPSPP.

Non-canonical homoterpenes have been identified in nature, and include the plant semiochemicals C11 (*E*)-4,8-dimethyl-1,3,7-nonatriene (DMNT) and C16 C(*E*,*E*)-4,8,12-trimethyltrideca-1,3,7,11-tetraene (TMTT),^138^ C11 2-methylisoborneol (Fig. 15A),^139^ C16 sex-pheromones 9-methylgermacrene B^140^ and 3-methyl-α-himachalene^141^ from the sandfly *Lutzomyia longipalpis*, longestin from *Streptomyces argenteolus*,^142^ C16 sodorifen from *Serratia plymuthica*,^143^ and C17 chlororaphens A and B from *Pseudomonas chlororaphis*.^144,145^ Incorporation of methyl groups is typically accomplished through *S*-adenosylmethionine (SAM) dependent methyltransferases to prenyl pyrophosphate substrates prior to cyclisation. In certain examples methyltransferases also initiate carbocation formation and cyclisation of terpenes.^25^ For instance, methylation induced cyclisation of FPP in bacteria produces the C16 intermediate α-presodorifen pyrophosphate (α-PSPP) (Fig. 15C).^143^ Further cyclisation of α-PSPP through terpene synthases yields diverse C16 polycyclic terpenes,^146^ including sodorifen which forms through an unusual ionisation induced fragmentation and recombination mechanism.^147^

Exploiting methyl transferases using synthetic biology approaches has enabled direct methylation of GGPP, FPP,^143^ GPP, DMAPP,^148^ IPP^149^ and other substrates with different regio-, and stereo-selectivity. Like the chemoenzymatic synthesis of homoterpenes from chemically produced precursors, these building blocks can be assembled into non-canonical terpenes. For instance, IPP methyltransferase from *Streptomyces monomycini* catalyses the formation of (*E*)-4-methyl-IPP and to a lesser degree (*Z*)-4-methyl-IPP which were used *in vivo* to synthesise new carotenoids.^150^ DMAPP analogues with methylation at the 2, 4, and 5 carbon positions have all been extended to non-canonical terpene products (Fig. 15A and B).^148,149^ The *S. plymuthica* sodorifenyl methyltransferase and variants were used to produce C16 α-PSPP and analogues and combined with terpene synthases to produce a library of novel C16 products (Fig. 15C).^151^ Engineering methyltransferases has been effective at further altering substrate scope and methylation positions.^148^ The only limitation is that enzyme promiscuity often results in multiple products and isomerisation of analogues. For sterol methyltransferase from *Chlamydomonas reinhardtii* a variant was engineered which installed a methyl group to the 10 position of farnesol but also shifted the terminal double bond from C10–C11 to C11–C12 to produce 10-methyl-11-ene-(*E,E*)-farnesol.^152^

## 4. Biocatalytic production of terpenes and their analogues using TPSs

Terpene production either *in vivo* by heterologous production or by biocatalysis using isolated enzymes relies on the efficiency of terpene synthase catalysis. Screening of terpene synthases can significantly increase product titres/yields.^58,153,154^ The catalytic efficiency of terpene synthases is poor relative to other enzyme classes. Typical catalytic rates (*k*_cat_) are often only 10^−2^–10^−5^ s^−1^ and reaction total turnover numbers (TTNs) are low.^52,53,65,74^ The presteady state catalytic rate is however significantly faster. Trichodiene synthase presteady state rate is approximately 40-fold larger than its steady state rate at 3.5–3.8 s^−1^.^155^ Catalysis is instead limited by product release as terpenes are poorly soluble in aqueous environments. Typically, both biocatalytic reactions and heterologous cultures are set up as biphasic mixtures with a hydrocarbon overlay.^156^ The rate is then limited by the mass-transfer between aqueous and organic phases. For typical multiple day biocatalytic transformation using isolated enzymes yields only reach ∼10–30%.^157,158^

### 4.1. Biphasic flow systems for terpene catalysis

**Fig. 16:**
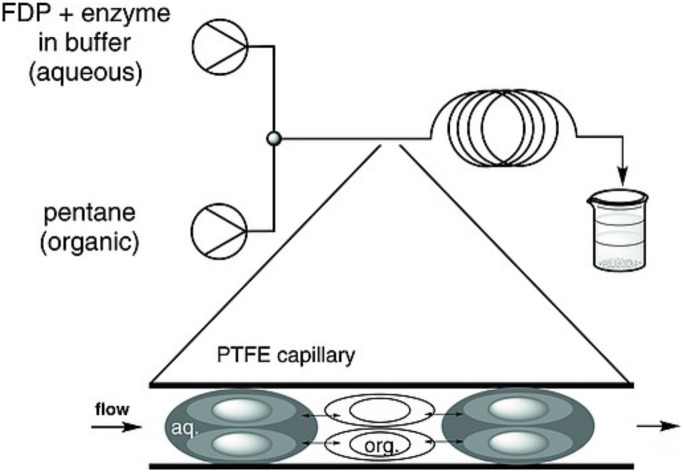
Principle of biphasic continuous flow biocatalysis for terpene production. Figure was reproduced from ref. 157 under the CC BY 4.0 licence.

To improve terpene biocatalysis, we established a biphasic continuous flow method by pumping aqueous and organic solutions through a T-junction to generate alternating segments of each phase (Fig. 16).^159^ Pentane (Log *P* of 3.4) was identified as the best solvent with minimal enzyme inactivation.^157^ The reactor internal diameter, which impacts both the segment size, surface area and the shear forces which drive internal mixing within the segments, was optimised. For the sesquiterpene synthases PR-AS, DCS and GDS the biphasic system increased productivity by 2-3-fold and achieved yields greater than 50%.^157,159^ These methods were then used successfully to produce amorpha-4,11-diene using ADS with 69% yield as a precursor for artemisinin.^157^ Analogues 12-methyl, 14-methyl and 15-methylFPP were also converted to the corresponding products using segmented flow methods with improved yields.

High-performance counter current chromatography (HPCCC) was subsequently used to improve biphasic continuous flow biocatalysis and reduce segment size.^158^ For PR-AS HPCCC established >95% yields and reduced the reaction time to 11 minutes; ∼10× faster than previous biphasic methods. Importantly the HPCCC reaction readily scaled allowing isolated yields of 70–94% totaling ∼30 mg for several terpene products. The strategy also improved the yield of dihydroartemisinic aldehyde by 3-fold with ADS from the 12-hydroxyFPP analogue when compared with batch reactions. Enzymatic TTN were improved in all cases using HPCCC and ranged from 54–110 for sesquiterpene synthases with FPP; poor compared with other enzyme classes but significantly improved compared with large-scale biocatalytic batch reactions.

### 4.2. Immobilisation of terpene synthases

To improve efficiency of using isolated enzymes we also attempted to immobilised terpene synthases on to a solid support using the N-terminal histidine tag so that the enzymes could be reused.^160^ As typical of immobilisation technologies, the enzyme activity was reduced by ∼20% but the enzymes were easily reused to increase productivity. In this case, immobilised Gd11olS was reused six times giving ∼60% yield and a TTN of ∼16. The matrix was loaded onto a packed bed reactor and a biphasic continuous flow method established with immobilised enzyme. Both Gd11olS and Gd4olS delivered ∼50% yields for 50–70 cycles and up to 19 hours. In this case, the TTN (2–3) was reduced although the reaction could have been continued at similar productivity. Compared to previous methods immobilised enzyme reaction rates were reduced most likely due the act of immobilisation impacting mass transfer of the product from the enzyme within the matrix to the organic phase. The advantage is that the immobilised enzyme was stable for extended periods with organic solvents.

### 4.3. Application of biphasic flow methods

Biphasic flow/HPCCC methods may be useful for extraction of *in vivo* cultures for production of terpenes as well as for biocatalysis. The obvious advantage of biophasic flow systems is that the surface area between aqueous and organic phases is increased improving extraction of terpene products. These methods could also help reduce the required volume of solvent to improve sustainability and costs. One limitation is that flow systems can result in emulsions and due to the length and diameter of the reactor can end in blockages. Nonetheless we have demonstrated the technology successfully for biocatalysis which improves the productivity of terpene synthases for both natural substrates and analogues.

## 5. Conclusions

Harnessing terpenoids for medicine, agriculture and other applications typically requires discovery of natural products and the biosynthetic pathway and genes associated with bioproduction. Mining terpene synthases from genomes is however time-consuming, requires experimental validation and ends with significant redundancy of terpene products. For instance, there are numerous germacrene A synthases reported from different bacterial and plant sources.^161–163^ In addition, not all terpene synthases from nature are applicable for scaled production and can suffer from low fidelity and activity. Engineering terpene synthase chemistry to produce new products is an attractive alternative. We and others have shown that combined computational and experimental approaches are useful to manipulate water capture in terpene synthases.^51,55,64,96^ Simulation-guided engineering using MD simulations is effective at establishing substrate binding modes, key active site residues involved in substrate folding and location of active site water. Methods establish enzyme variants for experimental testing that target nucleophilic water attack, and which do not end in acyclic products. ‘Hotspots’ within the active site including the G1/2 helix break, K and H helices have been repeatedly identified to be involved in water interactions. When mutated these sites can alter both the mechanism of cyclisation and final quenching of the carbocation or deprotonation steps. There are likely conserved regions within terpene synthases where water can enter the active site. Active site water alone does not however necessarily result in hydroxylated terpene products. When engineering water capture, variants still need to be experimentally tested. Successes are likely to accumulate neutral intermediates or products which deprotonate/hydroxylate prematurely. Understanding the reaction coordinate is vital for predictive engineering. In our experience, even small number of variants can give noteworthy results that alter water capture to produce hydroxylated or non-hydroxylated products. Optimisation of activity and catalytic efficiency of these initial variants remains limited. Medium-throughput screening methods are available with GC-MS and other techniques but improved high-throughput methods with genetic sequence and terpene product information retained would be valuable.^58,164,165^

Terpene synthase substrate promiscuity opens terpene chemistry to novel products derived from substrate analogues. In general substrates with altered double bond positions, additional methyl groups and chain length variations are readily accepted by terpene synthases. Heteroatoms substitutions can also be successful incorporated although care over position and choice of function group is necessary. The impact on cyclisation and product distributions of substrate analogues depends on the substrate and terpene synthase in question. If the chemical change in the substrate participates directly in the carbocation reaction cascade, then new products are likely. In certain cases, this delivers chemical novelty not possible for natural substrates. Rational engineering is challenging and linking novel compounds directly to applications is rarely performed. We show that the approach can be useful for obtaining new molecules with altered olfactory responses that could have applications for controlling insect pests in agriculture and disease vectors for human health.^67^

To translate new terpene chemistry into applications both product distribution and enzyme activity must be optimised. Biocatalytic terpene production suffers from low enzyme productivity because of inefficient product release into the aqueous solvent. Biphasic flow methods increase efficiency and can be used with valuable substrate analogues.^157,158^ Combining our knowledge of terpene synthase chemistry with high-throughput activity screens linked to production and application testing is essential to fully exploit terpene synthases in the future. We see the field converging on multidisciplinary methods that include generating novel terpene products, engineering terpene synthase and terpene modifying enzymes, developing high yielding production, and testing for novel applications.

## Author contributions

Luke A Johnson: conceptualization (lead); writing – original draft (lead); review and editing (equal). Rudolf K Allemann: review and editing (equal).

## Data availability

No primary research results, software or code have been included and no new data were generated or analysed as part of this review.

## Conflicts of interest

RKA currently holds a patent for analogues of (S)-germacrene D for use as insect repellents or attractants, EP3247799A1, US10334845B2. There are no other conflicts to declare.
